# Higher pressure differentiation of arc magmas under thicker crust

**DOI:** 10.1093/nsr/nwac278

**Published:** 2022-12-07

**Authors:** Yi-Gang Xu

**Affiliations:** State Key Laboratory of Isotope Geochemistry, Guangzhou Institute of Geochemistry, Chinese Academy of Sciences, China; Southern Marine Science and Engineering Guangdong Laboratory (Guangzhou), China

The formation of continental crust is pivotal to understanding the evolution of the earth and its habitability. The average intermediate or ‘andesitic’ bulk composition and characteristic depletion of niobium of the continental crust are strikingly matched by convergent margin magmas [1]. This has led to the inference that most continental crust is derived from igneous, arc crust. However, continental crust has characteristically higher Mg# (>0.5) than most oceanic arc andesites (<0.5). Therefore, an intriguing question to be answered in igneous petrology is which process was responsible for increasing Mg# of arc magmas? At least three principle models have been put forward, including intracrustal magmatic differentiation of primitive arc basalt or andesite, melt-peridotite interaction and magma recharge/mixing [2]. This long-lasting debate has recently been revived by Tang *et al*. [3], who compared global data of andesitic arc lavas emplaced both in continental and oceanic settings. They found that andesites from mature continental arcs with crustal thickness >45 km have systematically higher Mg# than those from oceanic arcs with crustal thickness <30 km. The elevated Mg# in continental arc lavas is attributed to strong Fe depletion as a result of garnet (± amphibole) fractionation, because the fractionation level in continental arcs is deeper than in oceanic arcs. Tang *et al*. [3] negated the need to invoke the other two hypothesized processes as the dominant mechanism to explain the high Mg# of most continental arc magmas and the continental crust, because of relatively limited occurrence of high magnesian andesite/adakites, and lack of any correlation between andesite Mg# and subduction thermal parameter.

The results of Tang *et al*. [3] yield important implications regarding the formation and evolution of continental crust. Because of preferential occurrence of Fe depletion of calc-alkaline magmas in thick-crust arcs, synmagmatic crustal thickening is considered as the key to continental crust formation. Moreover, the contrasting thickness between the crust at the time of formation and the present-day thin crust lends support to the idea that extensive thinning may have taken place after the formation of the continental crust, possibly due to lower crust delamination [1] and erosion.

While Tang *et al*. [3] provided a first-order, plausible explanation for the relatively high Mg# of continental arc magmas and hence of continental crust, uncertainties remain, largely associated with data treatment and simplified assumptions. The analyses of complied data were proceeded in a way of simple averaging. For instance, Figure 1c in Ref. [3] shows a roughly linear correlation of averaged Mg# of arc lavas with crustal thickness (<50 km), however such a relationship is not obvious from bulk raw data. In fact, the actual meaning of the averaged Mg# of thousands of arc lavas at a given crustal thickness remains obscure. Moreover, such treatment would average out any signals related to melt-rock interaction, magma mixing and different primary magmas, meaning that other competing possibilities for the generation of andesitic, continental crust with relatively high Mg# cannot be ruled out. Strictly speaking, since Tang *et al.* [3] focused on continental arc magmatism which was generated under a pre-existing continental crust, their results are more relevant to the evolution, *rather than* the origin, of continental crust. To avoid this confusion, data from thick oceanic arc should be evaluated too.

Despite these uncertainties, Tang *et al.* [3] provide a testable hypothesis for which a number of future studies are desirable. Variations of elements other than Mg# and Fe in continental calc-alkaline magmas should be compatible with the high-pressure magma differentiation model. For instance, if garnet fractionation is the primary driver in increasing Mg# of continental calc-alkaline magmas, the process would also concomitantly raise the La/Yb ratio and decrease Yb due to garnet being a major carrier of heavy rare earth elements. This can be tested by complementary and quantitative studies on the arc magmas and their related cumulates from the same arc. On the other hand, garnet-rich cumulates are expected to be present at thicker arc magmatic roots [4]. Although rare garnet pyroxenites have been reported at Kohistan and Talkeetna Arc [5], it remains to be clarified whether they represent igneous cumulates or metamorphosed phases of gabbro as a result of crustal thickening. For the arc regions where garnet cumulates are absent, high-resolution geophysical detection could be envisaged in order to trace potentially foundered garnet-rich arc roots.

In addition to fractionation pressure and water, oxygen fugacity is also crucial to Mg# of arc magmas, because the Fe^3+^/Fe_tot_ ratio, and therefore Mg#, of melts are strongly dependent on *f*_O2_ [6,7]. Given the considerable variation in *f*_O2_ in continental and oceanic arcs as well as in the course of magmatic fractionation, oxygen fugacity is necessary to be taken into account in deciphering differentiation processes of calc-alkaline magmatism.
